# Role of Endoscopic Ultrasonography and Endoscopic Retrograde Cholangiopancreatography in the Diagnosis of Pancreatic Cancer

**DOI:** 10.3390/diagnostics11020238

**Published:** 2021-02-04

**Authors:** Yasutaka Ishii, Masahiro Serikawa, Tomofumi Tsuboi, Ryota Kawamura, Ken Tsushima, Shinya Nakamura, Tetsuro Hirano, Ayami Fukiage, Takeshi Mori, Juri Ikemoto, Yusuke Kiyoshita, Sho Saeki, Yosuke Tamura, Sayaka Miyamoto, Kazuaki Chayama

**Affiliations:** 1 Department of Gastroenterology and Metabolism, Graduate School of Biomedical & Health Sciences, Hiroshima University, 1-2-3 Kasumi, Minami-ku, Hiroshima 734-8551, Japan; serikawa@hiroshima-u.ac.jp (M.S.); tsuboitomo@hiroshima-u.ac.jp (T.T.); ryokawa@hiroshima-u.ac.jp (R.K.); kenken0121@hiroshima-u.ac.jp (K.T.); d160174@hiroshima-u.ac.jp (S.N.); techan@hiroshima-u.ac.jp (T.H.); ayami@hiroshima-u.ac.jp (A.F.); tmori@hiroshima-u.ac.jp (T.M.); juri1118@hiroshima-u.ac.jp (J.I.); kiyoshi825@hiroshima-u.ac.jp (Y.K.); sho9@hiroshima-u.ac.jp (S.S.); yotamura@hiroshima-u.ac.jp (Y.T.); msayaka3@hiroshima-u.ac.jp (S.M.); chayama@mba.ocn.ne.jp (K.C.); 2 Research Center for Hepatology and Gastroenterology, Hiroshima University, 1-2-3 Kasumi, Minami-ku, Hiroshima 734-8551, Japan; 3 RIKEN Center for Integrative Medical Sciences, 1-7-22 Suehiro-cho, Tsurumi-ku, Yokohama, Kanagawa 203-0045, Japan

**Keywords:** pancreatic cancer, endoscopic ultrasonography, endoscopic retrograde cholangiopancreatography, early diagnosis

## Abstract

Pancreatic cancer has the poorest prognosis among all cancers, and early diagnosis is essential for improving the prognosis. Along with radiologic modalities, such as computed tomography (CT) and magnetic resonance imaging (MRI), endoscopic modalities play an important role in the diagnosis of pancreatic cancer. This review evaluates the roles of two of those modalities, endoscopic ultrasonography (EUS) and endoscopic retrograde cholangiopancreatography (ERCP), in the diagnosis of pancreatic cancer. EUS can detect pancreatic cancer with higher sensitivity and has excellent sensitivity for the diagnosis of small pancreatic cancer that cannot be detected by other imaging modalities. EUS may be useful for the surveillance of pancreatic cancer in high-risk individuals. Contrast-enhanced EUS and EUS elastography are also useful for differentiating solid pancreatic tumors. In addition, EUS-guided fine needle aspiration shows excellent sensitivity and specificity, even for small pancreatic cancer, and is an essential examination method for the definitive pathological diagnosis and treatment decision strategy. On the other hand, ERCP is invasive and performed less frequently for the purpose of diagnosing pancreatic cancer. However, ERCP is essential in cases that require evaluation of pancreatic duct stricture that may be early pancreatic cancer or those that require differentiation from focal autoimmune pancreatitis.

## 1. Introduction

The number of patients with pancreatic cancer and their mortality is steadily increasing [1,2]; with a five-year survival rate of less than 10%, pancreatic cancer has the worst prognosis among all cancers. This is because many patients with pancreatic cancer are diagnosed at an advanced stage, such as with metastasis, but the five-year survival rate for patients with Union for International Cancer Control (UICC) stage 0 (in situ) is 85.8%, and that for patients with stage IA (tumor with a maximum diameter 20 mm or less localized in the pancreas and no lymph node metastasis) is 68.7%, with a relatively good prognosis [3]. Therefore, it is essential to make a diagnosis at an early stage to improve the prognosis of pancreatic cancer.

Contrast-enhanced computed tomography (CT) and magnetic resonance imaging (MRI) are useful for diagnosing pancreatic cancer, and in Japanese clinical practice guidelines for pancreatic cancer [4], they are positioned as the first modality to be performed in patients with suspected pancreatic cancer based on clinical symptoms, such as abdominal pain, serum pancreatic enzymes, tumor markers, and transabdominal ultrasonography (US). Although endoscopic ultrasonography (EUS) and endoscopic retrograde cholangiopancreatography (ERCP) are invasive examination methods, they are important in the diagnosis of pancreatic cancer. EUS can obtain ultrasonic images of the pancreas in close proximity to the pancreas by scanning the stomach or duodenum, with very high spatial resolution. In addition, it is possible to obtain a pathological diagnosis by EUS-guided fine needle aspiration (EUS-FNA). ERCP allows for the detailed evaluation of pancreatic duct images, followed by pancreatic juice cytology.

This review aimed to evaluate the role of EUS and ERCP in the diagnosis of pancreatic cancer.

## 2. EUS

### 2.1. Diagnostic Performance of EUS for Pancreatic Cancer

**Figure 1:**
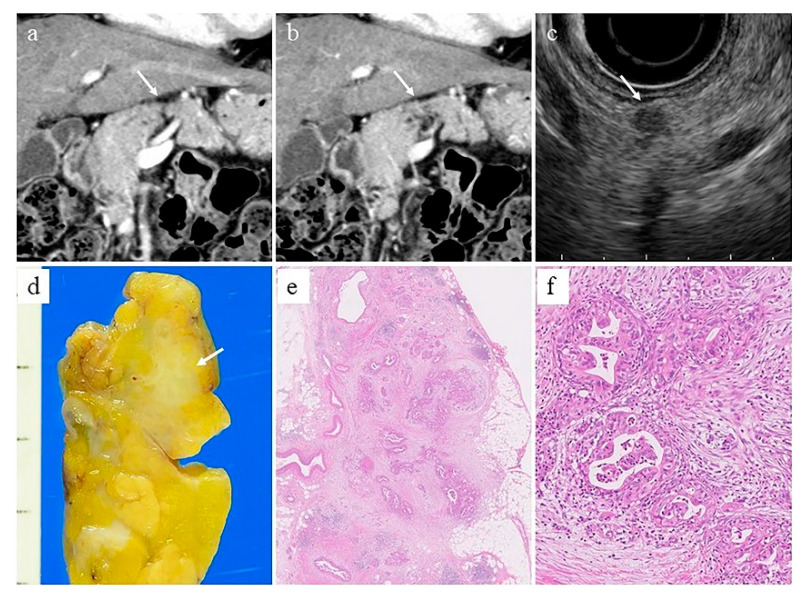
A 68-year-old woman with small pancreatic cancer. (**a,b**) Computed tomography shows parenchymal atrophy of the pancreatic body (arrow), but no obvious mass. (**c**) Endoscopic ultrasonography shows a well-defined, irregular hypoechoic mass with a diameter of 8 mm in the pancreatic body (arrow). (**d**) Surgically resected specimen shows a grayish-white solid mass (arrow). (**e**) Loupe image of the mass. (**f**) Moderately differentiated tubular adenocarcinoma (magnification: ×100).

EUS is a very sensitive imaging modality for detecting pancreatic cancer, although it is more invasive and operator-dependent than CT and MRI. In a systematic review of nine studies and 678 patients, Dewitt et al. [5] reported that EUS was more sensitive than CT for the detection of pancreatic cancer (91–100% vs. 53–91%). Similarly, in a systematic review of 22 studies and 1170 patients, Kitano et al. [6] reported that EUS was more sensitive than US and CT for the detection of pancreatic tumors (94% vs. 67% and 98% vs. 74%, respectively). In addition, EUS is very useful for the detection of small pancreatic cancers (Figure 1). The detection rate of small pancreatic cancer of 2 cm or less by contrast-enhanced CT has been reported to be 50–77% [7,8,9,10,11,12], which is by no means a good result. On the other hand, EUS has been reported to be better than contrast-enhanced CT, with a sensitivity of 80–100% for detecting small pancreatic cancer of 2 cm or less [7,8,9,11,12]. In one meta-analysis, EUS was reported to show a pooled sensitivity of 85% for the detection of pancreatic tumors that could not be detected by multi-detector row CT (MDCT) [13]. Yasuda et al. [14] reported that in 132 patients with elevated tumor markers, elevated serum amylase, or dilated main pancreatic duct, EUS was able to detect pancreatic cancer smaller than 10 mm that could not be detected by contrast-enhanced CT in three patients.

EUS is also useful for staging pancreatic cancer, i.e., for assessing vascular invasion and lymph node metastasis. Two meta-analyses of vascular invasion reported that the pooled sensitivity of EUS was 85% and 72%, and the pooled specificity was 91% and 89%, respectively, with higher sensitivity and equivalent specificity compared to CT [15,16]. Moreover, in a meta-analysis of nodal staging [15], the pooled sensitivity and specificity of EUS were 69% and 81%, respectively, and compared to CT, the pooled sensitivity was high (58% vs. 24%) and pooled specificity was equivalent (85% vs. 88%).

### 2.2. EUS for Differential Diagnosis of Pancreatic Cancer

The utility of contrast-enhanced EUS (CE-EUS) and EUS elastography in the contemporary diagnosis of solid pancreatic masses has been reported.

CE-EUS enables the acquisition of contrast images of peripheral vessels and perfusion images of parenchymal organs by imaging a nonlinear signal obtained by resonating a contrast agent containing microbubbles with low acoustic pressure. In CE-EUS, the presence or absence of blood flow and enhancement patterns are useful for the differential diagnosis of solid pancreatic masses [12,17,18,19,20,21,22,23,24,25]. In other words, pancreatic cancer is heterogeneously enhanced in the lesion and exhibits a hypovascular pattern as compared with the peripheral pancreatic parenchyma, and many focal pancreatitides are homogeneously enhanced and exhibit an isovascular to hypervascular pattern as compared with the peripheral pancreatic parenchyma, and many neuroendocrine tumors and pancreatic metastases of renal cell carcinoma exhibit a hypervascular pattern. Furthermore, in CE-EUS, it is also possible to draw a time intensity curve (TIC) that graphs the change over time in echo intensity due to contrast enhancement. The utility of quantitative evaluation of enhancement patterns using TICs in the differential diagnosis of pancreatic masses has been reported [23,26,27,28,29]. In four meta-analyses, the pooled sensitivity and specificity of CE-EUS in the diagnosis of pancreatic cancer were 91–93% and 80–88%, respectively [30,31,32,33]. In addition, Yamashita et al. [34] reported that the sensitivity of CE-EUS for the detection of pancreatic cancer in small pancreatic masses of 11–20 mm was significantly better than that of MDCT and MRI (95% compared to 78%, and 73%, respectively), and that the sensitivity of CE-EUS in pancreatic masses of 10 mm or less was also significantly better than that of MDCT (70% and 20%, respectively).

Elastography is a technology for imaging tissue elasticity information using ultrasonography, and it includes strain elastography and shear wave elastography. Strain elastography is a technology for imaging the relative degree of tissue strain obtained by probe compression or heartbeat. Pixel values within the region of interest were converted to color images within the range of 0 (red) for minimum hardness to 255 (blue) for maximum hardness, which created a distribution histogram. Reported evaluation methods by strain elastography include a qualitative evaluation method using color pattern [35,36,37,38,39] and a quantitative evaluation method measuring strain ratio, strain histogram, and neural network [22,25,40,41,42,43,44,45]. On the other hand, shear wave elastography can measure the absolute value of tissue hardness by calculating the propagation velocity of shear waves and Young’s modulus. Shear wave elastography was introduced to EUS, but Ohno et al. [46] reported that conventional strain ratio and strain histogram measurements were superior in the diagnosis of pancreatic masses. In a meta-analysis [47], the pooled sensitivity and specificity of EUS elastography in the diagnosis of malignant pancreatic tumors were reported to be 98% and 63% by the qualitative evaluation method, and 95% and 61% by the quantitative evaluation method, respectively.

### 2.3. Surveillance of Pancreatic Cancer For High-Risk Individuals

An individual with a strong family history of pancreatic cancer or a hereditary syndrome in which pancreatic cancer is a phenotypic symptom is defined as a high-risk individual (HRI) [48], and surveillance aimed at the early detection of pancreatic cancer is recommended. HRIs include the following patients [49]: individuals who have at least one first-degree relative with pancreatic cancer who in turn also have a first-degree relative with pancreatic cancer; all patients with Peutz-Jeghers syndrome; all carriers of a germline *CDKN2A* mutation; and carriers of a germline *BRCA*, *BRCA1*, *PALB2*, *ATM*, *MLH1*, *MSH2*, or *MSH6* gene mutation with at least one affected first-degree blood relative. MRI/magnetic resonance cholangiopancreatography (MRCP) and EUS are recommended as diagnostic imaging modalities, considering cumulative radiation exposure from frequent CT [49]. Canto et al. [50] reported that in 216 asymptomatic HRIs, focal pancreatic abnormalities were found in 92 patients (42.3%) during an average follow-up period of 28.8 months (one or more cystic lesions in 84 patients, solid lesions in three patients, and isolated dilated main pancreatic duct in five patients), and the detection rate of lesions with CT, MRI, and EUS was 13.8%, 77%, and 79%, respectively. Harinck et al. [51] also reported that 11 mm and 7 mm solid lesions and nine cysts of 10 mm and above were found in nine patients (6%) in a comparative prospective trial of EUS and MRI detection rates for clinically relevant lesions at the first screening for 139 asymptomatic HRIs. In their study, both solid lesions were detected only by EUS (one with stage I pancreatic cancer and the other with multifocal pancreatic intraepithelial neoplasia 2), and three cysts (33%) were detected only by MRI. EUS, which can detect even small lesions, is considered optimal for the surveillance of pancreatic cancer in HRIs. However, it has also been reported that there is low consensus among observers on the interpretation of EUS in HRIs [52]; therefore, complimentary examination in combination with MRI is recommended.

### 2.4. EUS-FNA

Pathological diagnosis is required to determine the treatment strategy for pancreatic cancer, and EUS-FNA is the first-line diagnostic method. In several meta-analyses of EUS-FNA for solid pancreatic lesions [53,54,55,56], the pooled sensitivity and specificity were 85–92% and 96–98%, respectively, and its usefulness as a pathological diagnostic method has been proven. Regarding lesion size, the accuracy of EUS-FNA has been reported to be 93.4% for lesions ≥20 mm, 83.5% for 10–20 mm lesions, and 82.5% for lesions that are 10 mm or less [57]. In addition, the sensitivity and specificity of EUS-FNA for the evaluation of para-aortic lymph node metastasis in patients with pancreatobiliary carcinoma have been reported to be 96.7% and 100%, respectively, which are better than those of positron emission tomography with CT [58]. Factors that affect diagnostic performance include the type and diameter of FNA needles, suction method, and the presence or absence of rapid on-site evaluation (ROSE). Biopsy needles with a characteristic shape have been used for tissue collection (e.g., Acquire^TM^ (Boston Scientific Corp., Marlborough, MA, USA) and EchoTip ProCore^®^ (COOK Medical, Bloomington, IN, USA)). The tissue collection method using these needles is called EUS-guided fine-needle biopsy (EUS-FNB). Several meta-analyses have compared the diagnostic performance and sample collection rate based on the sample collection methods (EUS-FNA or EUS-FNB) and needle diameters; however, no consensus has yet been reached [59,60,61,62]. In a meta-analysis [63], ROSE was reported to have no effect on the diagnostic yield, collection rate of adequate samples, pooled sensitivity, and pooled specificity.

There has been a contemporary movement to perform genomic profiling of pancreatic cancer by next-generation sequencing (NGS), and apply it to treatment selection. The efficacy of platinum agents for pancreatic cancer with a germline *BRCA/PALB2* mutation and of olaparib, a poly (ADP-ribose) polymerase (PARP) inhibitor, as maintenance therapy after first-line platinum-based chemotherapy for pancreatic cancer with germline *BRCA1/2* have been shown [64,65]. In addition, although the frequency is extremely low in pancreatic cancer, therapeutic agents for advanced solid tumors with high-frequency microsatellite instability (MSI) and neurotrophic tyrosine receptor kinase (*NTRK*) gene fusions have been clinically introduced, including pembrolizumab as an immune checkpoint inhibitor and entrectinib as an *NTRK* inhibitor [66,67]. According to the National Comprehensive Cancer Network (NCCN) guideline 2020 [68], germline testing, tumor/somatic gene profiling, MSI, and/or mismatch repair testing is recommended, if not previously performed, for pancreatic cancer with distant metastasis and locally advanced pancreatic cancer. Tissue samples used for these tests are often collected by EUS-FNA/FNB, and the collection rate of samples suitable for these tests has been reported [69,70,71,72,73]. In these studies, the collection rate of samples suitable for NGS was 70–97%, and EUS-FNB performed better.

EUS-FNA is a very safe procedure, and a prospective multicenter study has reported that the complication rate of EUS-FNA for solid pancreatic masses was 1.2% [74]. On the other hand, there have been some case reports on needle tract seeding by EUS-FNA [75], which is a complication that cannot be overlooked in patients where radical resection is possible. Yane et al. [76] reported that the incidence of needle tract seeding after EUS-FNA was 3.4% in patients with pancreatic cancer who underwent distal pancreatectomy.

## 3. ERCP

### 3.1. Diagnostic Performance of ERCP for Pancreatic Cancer

With the progress of imaging modalities, such as CT, MRI, and EUS, and the widespread use of EUS-FNA as a pathological diagnostic method, the chance of performing ERCP for the purpose of diagnosing pancreatic cancer is decreasing. Therefore, ERCP is mainly performed as a therapeutic procedure, such as biliary drainage for bile duct stricture due to pancreatic head cancer. The sensitivity of pancreatic juice cytology, including brushing cytology in the diagnosis of pancreatic cancer, is reported to be 21.3–63.6% [77,78,79,80,81,82], which is far below the diagnostic performance of EUS-FNA. On the other hand, because most pancreatic cancers are invasive ductal adenocarcinomas that develop from the pancreatic duct epithelium, some changes in the pancreatic duct image are observed in most patients. In a meta-analysis of the diagnostic performance of pancreatic cancer by evaluation of pancreatic duct images by endoscopic retrograde pancreatography (ERP), the pooled sensitivity and specificity were reported to be 57.9% and 90.6%, respectively [83]. Some early-stage pancreatic cancers (Stage 0, I) cannot be detected as tumors by CT, MRI, or EUS, and it is difficult to collect samples by EUS-FNA. Especially for stage 0 pancreatic cancer, which is carcinoma in situ, localized main pancreatic duct stricture is often the only image finding, and detailed evaluation of the pancreatic duct image by ERP and subsequent pancreatic juice cytology are extremely important for the diagnosis. It has been reported that detailed evaluation of pancreatic duct images, including branch pancreatic ducts by endoscopic balloon catheter spot pancreatography, was useful for the diagnosis of small pancreatic cancer [84]. In addition, the usefulness of serial pancreatic juice cytology using an endoscopic nasopancreatic drainage catheter in the diagnosis of pancreatic carcinoma in situ has been reported, and the sensitivity was excellent at 72.2–100% [85,86,87]. On the other hand, ERCP has the problems of high invasiveness and complications related to acute pancreatitis. In several cohort studies, the incidence of acute pancreatitis due to diagnostic ERCP has been reported to be 0.7–11.8% [88,89,90,91], and the incidence of post-ERCP pancreatitis limited to pancreatic cancer has been reported to be 3.6–11.5% [86,92].

### 3.2. Differential Diagnostic from Autoimmune Pancreatitis

**Figure 2:**
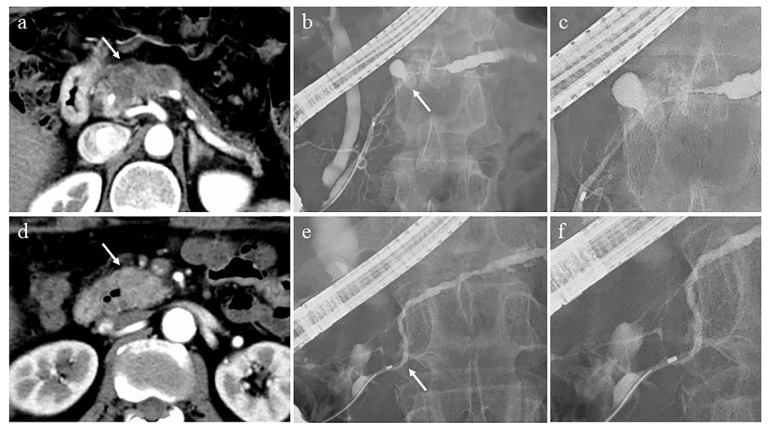
Cases of pancreatic cancer (**a**–**c**) and autoimmune pancreatitis (**d**–**f**). (**a**) Computed tomography (CT) shows an irregular and hypovascular 3 cm-seized mass in the pancreatic body (arrow) and dilatation of the upstream main pancreatic duct (MPD). (**b**) Endoscopic retrograde pancreatography (ERP) shows the MPD stricture in the pancreatic body (arrow) and upstream MPD dilatation. (**c**) Deviation of side branches from the stricture site is not observed. (**d**) CT shows a 2 cm-sized mass with hypovascularity in the arterial phase in the pancreatic head (arrow). (**e**) ERP shows mild strictures of the Wirsung and Santorini ducts (arrow), and slight upstream MPD dilatation. (**f**) Deviation of side branches from the stricture site is observed.

Evaluation of pancreatic duct images by ERP is important for the diagnosis of autoimmune pancreatitis (AIP) [93,94]. Diagnosis of AIP patients with diffuse pancreatic enlargement with a capsule-like rim [95] on CT or MRI is relatively easy and does not necessarily require ERP. On the other hand, AIP patients with atypical pancreatic parenchymal findings, such as focal pancreatic enlargement and mass formation, are difficult to distinguish from pancreatic cancer, and evaluation of the pancreatic duct image by ERP is important. The important ERP findings in the diagnosis of AIP are as follows [96]: long (> one-third the length of the pancreatic duct) stricture, lack of upstream dilatation from the stricture (<5 mm), multiple strictures, and side branches arising from the stricture site. In addition, it has been reported that the length of the narrowed portion of the main pancreatic duct (MPD) ≥ 3 cm, the side branches arising in the narrowed portion of the MPD, and the lack of upstream MPD dilatation from the narrowed portion were significantly more frequent findings in AIP than in pancreatic cancer, and useful for the differential diagnosis of AIP and pancreatic cancer [97,98] (Figure 2). MRCP is a simple and non-invasive imaging modality that is used to evaluate the pancreatic duct image of AIP. Its resolution is inferior to that of ERP [99], but it has the advantage of visualizing the upstream MPD when it is obstructed. MRCP images have been improved by advancements in MRI scanners and techniques, such as partial maximum intensity projection [100] and breath-hold compressed sensing accelerated three-dimensional MRCP [101]; MRCP findings have been added to the Japanese Clinical Criteria for Autoimmune Pancreatitis 2018 [94]. However, it is not possible to evaluate the derivation of the side branches arising from the narrowed portion of the MPD by MRCP, which is useful for the differential diagnosis of AIP and mass-forming pancreatitis from pancreatic cancer.

## 4. Conclusions

EUS can detect pancreatic cancer with higher sensitivity than CT and MRI and is useful for the early diagnosis of the disease. Furthermore, the addition of CE-EUS and EUS elastography can improve the accuracy of the differential diagnosis of pancreatic tumors. EUS has excellent sensitivity and specificity as a pathological diagnostic method for pancreatic cancer. Although ERCP is performed less frequently for the purpose of diagnosing pancreatic cancer, it is essential for the evaluation of pancreatic duct stricture that may be early pancreatic cancer or that requires differentiation from focal autoimmune pancreatitis. EUS-FNA, which enables relatively easy and safe tissue collection, plays a major role in precision medicine based on genomic profiling for advanced pancreatic cancer. In the future, multicenter, prospective studies are needed to clarify the optimal type of needles and suction method for collecting the amount of tissue required for genomic analysis.
